# Pattern of workplace violence against doctors practising modern medicine and the subsequent impact on patient care, in India

**DOI:** 10.1371/journal.pone.0239193

**Published:** 2020-09-18

**Authors:** Amandeep Kaur, Farhad Ahamed, Paramita Sengupta, Jitendra Majhi, Tandra Ghosh

**Affiliations:** 1 Department of Community Medicine & Family Medicine, All India Institute of Medical Sciences, Kalyani, West Bengal, India; 2 Department of Physiology, All India Institute of Medical Sciences, Kalyani, West Bengal, India; Institute of Economic Growth, INDIA

## Abstract

**Introduction:**

The incidents of violence against doctors, leading to grievous injury and even death, seem to be on an increasing trend in recent years. There is a paucity of studies on workplace violence against doctors and its effect, in India. The present study was conducted to assess workplace violence faced by doctors, its effect on the psycho-social wellbeing of the treating doctor and, subsequently, on patient management.

**Methods:**

The present nationwide cross-sectional study was conducted from November 2019 –April 2020. The sample size was calculated assuming the prevalence of workplace violence as 50%, with 20% non-response. Doctors, working in private and/or public set-up, with ≥1 year clinical experience, were included. A pre-tested study tool- Google form—was sent to study participants via social media platforms. The Microsoft Excel spreadsheet was downloaded from google drive and data was analysed using STATA-12 statistical software.

**Results:**

A total of 617 responses were received from doctors all over India; out of which 477 (77.3%) doctors had ever faced workplace violence. “Actual or perceived non-improvement or deterioration of patient’s condition" (40.0%), followed by “perception of wrong treatment given” (37.3%) were the main causes of workplace violence; and the family members/relatives were the major perpetrators (82.2%). More than half of the participants reported “loss of self-esteem”, “feeling of shame” and “stress/depression/anxiety/ideas of persecution” after the incident. Management by surgical interventions (p-value<0.001) and handling of emergency/complicated cases (p-value<0.001) decreased significantly with an increase in severity of workplace violence; while the suggestion of investigations and referrals increased (p-value<0.001).

**Conclusions:**

Workplace violence has a significant effect on the psycho-social well-being of doctors, as well as on patient management; which may escalate discontent and distrust among the general public, thereby increasing incidents of workplace violence—in a self-propagating vicious cycle.

## Introduction

Incidents, where employees are abused, threatened, assaulted, or subjected to offensive behaviour in circumstances related to their work, are defined as workplace violence (WPV) [1]. Workplace violence can occur in any organization, against anybody, and at any time. Healthcare workers such as physicians, nurses, and other providers (both community and hospital-based) are specifically vulnerable at the workplace due to direct contact with people; especially because they have to provide care to people in distress resounding great hope and expectation from the treating doctor.

Today workplace-related violence has become an inevitable part of healthcare, globally [2, 3]. The annual incidence of WPV is four times more in healthcare workers (8 serious cases per 10,000 full-time employees) as compared to all other professions (2 per 10,000 full-time employees) [4]. Unrealistic expectations of patients and relatives, like miraculous recovery from all disease conditions, is an important contributing factor. Although advanced medical care technology has revolutionized disease outcomes, factors like insufficient human resources, inefficient government policy, political pressure, poor infrastructure, commercialization of health care, exorbitant costs of tests and medicine, often compromise the provision of best possible care, despite best effort by the treating physicians [5, 6]. Such incongruity between high expectations and actual ground realities often spark-off anxiety and frustration among patients and their relatives, which inflicts anger and violence towards doctors. Moreover, recent advances in information technology have revolutionized access to healthcare-related information, which acts as a double-edged sword. Often, dubious internet-based information creates a concoction among patients' un-trained minds, which is detrimental for effective interaction between physicians and patients, and thereby hamper the building of a healthy doctor-patient relationship [7].

In recent times, intolerance and WPV against healthcare staff have emerged as a serious threat to medical fraternity resulting in tarnished reputation, grievous injuries, sometimes even death of the doctor [8, 9]. Indian Medical Association (IMA) has reported that 75% of doctors had experienced WPV, and 82.7% felt stressed out [10, 11]. Handling high demands and expectations, in a workplace environment vulnerable to violence, affect the psychological health of doctors [12]. The combined negative effects of violence and stress often cumulate exponentially and activate a vicious cycle which becomes very difficult to break [13].

Effect of violence on psycho-social health of doctors and its impact on critical decision making on patient’s management remains grossly under-researched and under-reported in India. Addressing these issues may provide solutions towards better doctor-patient relationships and thereby, better patient care. So far, most of the studies regarding WPV against physicians in India have been conducted among trainee doctors in single healthcare facilities, and none of them has addressed the consequences of WPV on patient management. This caveat warrants scientific documentation of WPV against doctors at the national level, which would be a guiding document for policymakers to formulate effective strategies for its prevention and control. In this study, a survey has been conducted to assess the type and frequency of WPV faced by doctors in varied setup (private and government healthcare facilities); and its impact on the management of patients in India.

## Methods

### Study setting

The present study was a nationwide internet-based study conducted by the Department of Community Medicine& Family Medicine, All India Institute of Medical Sciences (AIIMS), Kalyani, West Bengal, India. India is a constitutional republic consisting of 29 states, eight union territories; and Delhi the national capital territory. In India, providing healthcare to its citizens is the responsibility of the respective states. However, the majority of healthcare services are provided by the private sector [14].

### Study design

A cross-sectional study design was adopted for this study to collect information about the prevalence of workplace violence faced by doctors and its associated outcomes and exposures.

### Study period

The present study was conducted from November 2019 to April 2020.

### Study population

Doctors practising modern medicine in either private or public set-up or both, within India, formed the study population.

### Inclusion and exclusion criteria

Medical graduates (Bachelor of Medicine & Bachelor of Surgery, MBBS) and post-graduates (specialists and super-specialists) managing patients in a private or public healthcare setting with experience of more than one year were included in the study. Medical professionals who did not have an MBBS degree—doctors of other systems of medicine, nursing staff, and paramedics—but were managing patients in any type of healthcare setting were excluded from the study.

### Sample size

Assuming prevalence (p) of violence faced by practising doctors to be 50% and taking absolute allowable error (d) as 5%, the sample size was calculated using the formula (Z1-α)2p(1-p)/d2 at 95% confidence interval. The sample size thus calculated was 384. After adding a 20% non-response rate the final sample size was calculated to be 461.

### Study tool

A pre-tested semi-structured study questionnaire in the form of “Google form” was prepared. The study questionnaire included selected socio-demographic details, academic qualification, practice setting, details of violence faced, its psycho-social impact and effect on the management of patients. The survey questionnaire took approximately 10–15 minutes to fill depending upon the type of response (Annexure 1).

**Google forms** is an internet-based application developed by Google LLC. It allows collecting data/information from users via personalised surveys without levying any charges. Both open-ended and closed-ended questions can be included in the questionnaire which can further be customised as per the requirement of the survey being conducted. The form can be shared with respondents by sending the link through e-mail or WhatsApp message, who can fill the form by logging into their respective e-mail ids. The form can be filled using a computer, laptop, tablet, smart phone, or any electronic gadget supporting Google forms. The information thus collected is stored in the google drive of the investigator in the form of a spreadsheet.

Participant information and consent were inserted at the beginning of the google form itself. Moreover, filling and submission of the Google form were also taken as implied consent from the respondent.

The questionnaire was divided into three sections. ***Section one*** consisted of socio-demographic and professional details of the respondent. The question regarding facing WPV was kept at the end of Section one; the subsequent set of questions depended upon the positive or negative response to this question.

***Section two*** of the questionnaire consisted of questions regarding details of the type of violence faced and their alleged reasons, perpetrators of violence, reporting of the incident, and effect of violence on psycho-social well-being and patient management. A small description of the types of violence was given along with the options in the questionnaire itself, to prevent ambiguity in response. Trauma Screening Questionnaire (TSQ), which is freely available, the ten-item scale was inserted towards the end of Section two to assess the presence of post-traumatic stress of WPV. The said instrument has a sensitivity of 76%, specificity of 97%, and an overall efficiency of 92% among crime victims when the cut-off value of six is taken [15].

***Section three*** consisted of questions about professional satisfaction, workplace safety, the effect of the news on violence against doctors on patient management, and suggested interventions for the prevention of acts of WPV.

***Section one*** and ***three*** were made mandatory and required to be filled by each respondent irrespective of whether they had faced violence or not, while ***Section two*** was customised to be filled by only those respondents who had faced any type of WPV.

In the current paper, we have presented data of ***Section one*** and ***Section two*** only.

### Data collection

A message, briefly describing the purpose of the study, was formulated and the link of the Google form was embedded in the message. The signatures and affiliations of the investigators were added at the end of the message to establish credibility.

A preliminary list of e-mail addresses and WhatsApp numbers was made by the investigators from their contacts. Faculty members of other departments of AIIMS, Kalyani were also contacted, and contact details of all the doctors known to them was collected. The above-formulated message was then sent to the list of contacts via various social media platforms. As the response was suspected to be influenced by the inter-personal professional relations, other faculty members of the institute were also asked to forward the message along with a separate message to prompt the response and ensure reinforcement. The doctors to whom the message was sent were also requested to further circulate it among their colleagues from the medical fraternity. The study tool was customised in such a way that only one response per participant was possible. The e-mail address of the respondents was not collected to maintain anonymity and confidentiality. The filing of the form was voluntary. New messages were framed and recirculated at regular intervals to prompt the response.

The messages were circulated only in closed groups of medical fraternity so that non-medical persons cannot fake and fill the form.

The link to the Google form was de-activated once the desired sample size was reached. One week before deactivation of the link, a message containing the information regarding subsequent deactivation of the link was circulated among all the contacts.

### Operational definitions

- **Verbal abuse (VA):** Verbal abuse was defined as a perception of being professionally and personally attacked, devalued, or humiliated via the spoken word [16]. Verbal abuse included the use of abusive or offensive language, derogatory remarks, or profane and/or obscene comments.
- **Verbal Threat (VT):** Verbal threats were defined as warnings with the intent to injure doctor with or without an object or weapon, or to harass and/or to physically threaten them [17].
- **Intimidation (IN):** An act of frightening or threatening a doctor usually to pursue those to do something which the doctor did not want to do. This included issues like “issuing false certificate”, “issuing a false prescription to get Mediclaim benefits” etc.
- **Physical Injury (PI):** Physical violence was defined as an attack to create bodily injury. This included slapping, pinching, pushing, shoving, and spitting or kicking, with or without the use of weapons [17].
  - **Minor physical injury:** Physical injury required no or minimal medical attention.
  - **Major physical injury:** Physical injuries other than minor physical injury and requiring hospital admission.
- **Sexual abuse (SV):** Sexual abuse was defined as an unwelcome or uninvited action that involved sexual propositioning, sexual gesture, and physical contact of a sexual nature [16].
- **Damage to physical property (PD):** Physical injury to tangible property including resulting loss of use and loss of use of a tangible property that has not been physically injured.

### Statistical analysis

The automatically filled dataset in Microsoft Excel spreadsheet was downloaded from google drive of the investigator, coded and analysed with the help of STATA-12 statistical software. Categorical data were presented as percentages (%). Pearson’s Chi-square test was used to evaluate differences between groups for categorised variables; a p-value of less than 0.05 was considered as statistically significant.

To assess associations with socio-demographic, professional details, psycho-social wellbeing and patient management, the types of violence were grouped according to the severity of WPV faced–VA only, [(VT + IN) ± VA] and [(PI + SV + PD)±VA±(VT + IN)]. The study participants were labelled according to the greater severity of WPV faced, e.g., if the study participant has faced both verbal abuse and intimidation, he/she was counted under [(VT + IN) ± VA].

### Ethics

Ethical clearance was obtained from the Institutional Ethics Committee of All India Institute of Medical Sciences (AIIMS), Bhubaneswar, Odisha, India (Reference number–T/IM-NF/Kalyani/19/02).

## Results

**Fig 1:**
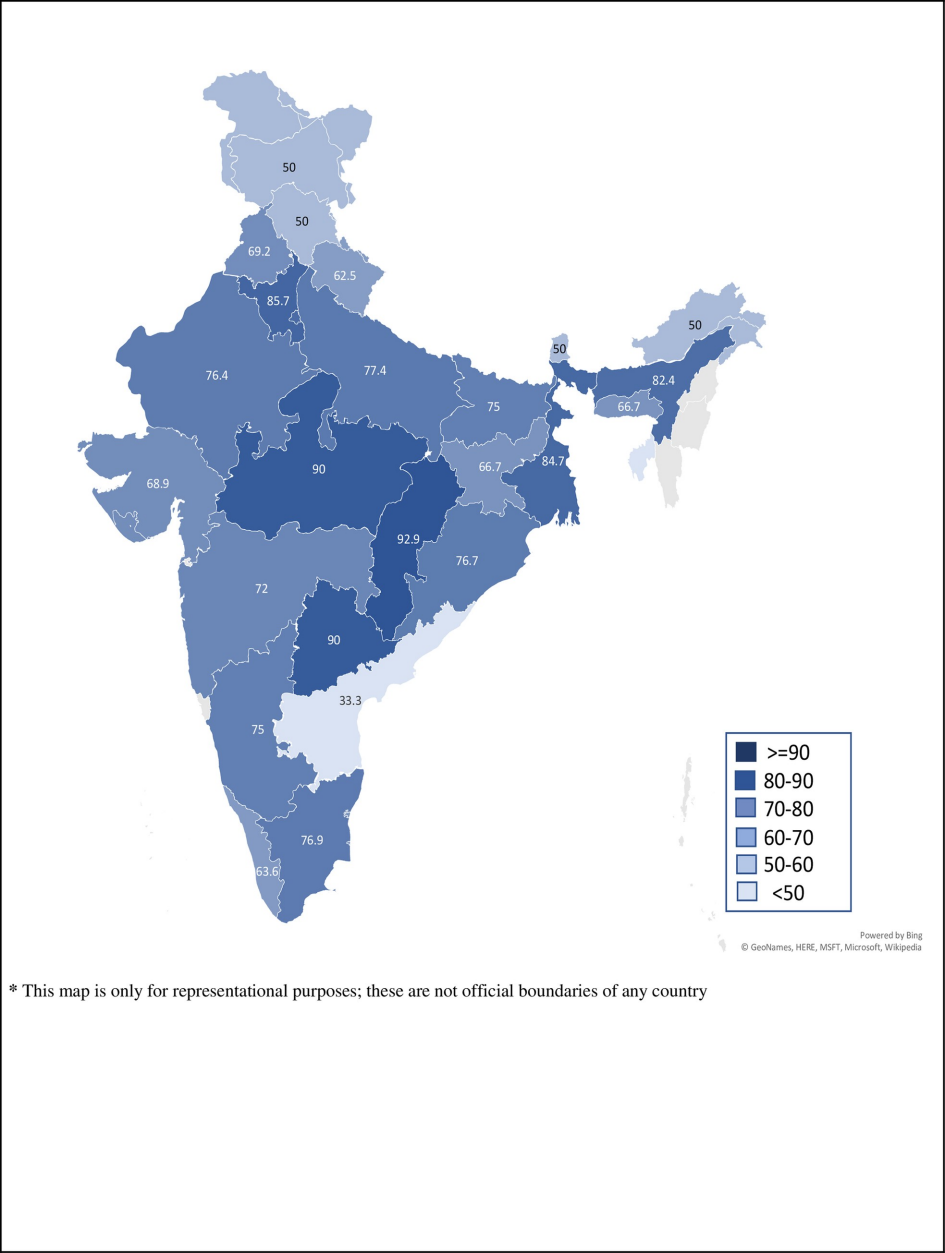
State-wise distribution of study participants who had ever-faced workplace violence. [(Total number of participants who faced workplace violence in the state / Total number of participants who responded from that state)*100].

We received responses from a total of 617 doctors; among them, 477 (77.3%) doctors had faced WPV at least once until the completion of the survey. The state-wise distribution of study participants who had ever-faced WPV, proportionate to the number of total responses from the respective states, is shown in Fig 1.

The mean (SD) age of Doctors who faced Work Place Violence (DWPV) was 37.9 (9.7) years; the maximum number of DWPV belonged to the age group of 31–40 years (41.7%). The chance of facing violence was found to be inversely related to the age of doctors. More male (78.3%) and unmarried (81.1%) DWPV in comparison to females (74.5%) and ever married (76.3%) doctors, respectively. Also, doctors practising in urban areas were seen to be more prone to WPV. However, there was no statistically significant difference between the socio-demographic and professional profiles of the doctors who faced workplace violence and those who did not face WPV (**Table 1**).

**Table 1:** Distribution of study participants as per socio-demography and professional details (n = 617).

S. no	Variables	Groups	Violence faced (D) Number (%)				Violence not faced (S) Number (%)				Total (D+S) Number (%) (N = 617)	p-value*
			Government (a) N = 189 N (%)	Private (b) N = 173 N (%)	Both (c) N = 115 N (%)	Total (a+b+c = D) N = 477 N (%)	Government (p) N = 55 N (%)	Private (q) N = 59 N (%)	Both (r) N = 26 N (%)	Total (p+q+r = S) N = 140 N (%)		
1.	**Age (years)**	**≤30**	90 (61.2)	14 (9.5)	17 (11.6)	**121 (82.3)**	20 (13.6)	4 (2.7)	2 (1.4)	**26 (17.7)**	**147 (100.0)**	**0.066**
		**31–40**	73 (28.5)	70 (27.3)	56 (21.9)	**199 (77.7)**	25 (9.8)	20 (7.8)	12 (4.7)	**57 (22.3)**	**256 (100.0)**	
		**41–50**	20 (14.4)	60 (43.2)	28 (20.1)	**108 (77.7)**	7 (5.0)	16 (11.5)	8 (5.7)	**31 (22.3)**	**139 (100.0)**	
		**51–60**	3 (6.4)	19 (40.4)	10 (21.3)	**32 (68.1)**	1 (2.1)	13 (27.6)	1 (2.1)	**15 (31.9)**	**47 (100.0)**	
		**>60**	3 (10.7)	10 (35.7)	4 (14.4)	**17 (60.8)**	2 (7.1)	6 (21.4)	3 (10.7)	**11 (39.2)**	**28 (100.0)**	
			*P<0*.*001*				*P<0*.*001*					
2.	**Gender**	**Female**	60 (36.4)	39 (23.6)	24 (14.5)	**123 (74.5)**	26 (15.8)	10 (6.1)	6 (3.6)	**42 (25.5)**	**165 (100.0)**	**0.188**
		**Male**	129 (28.5)	134 (29.6)	91 (20.1)	**354 (78.3)**	29 (6.4)	49 (10.8)	20 (4.4)	**98 (21.7)**	**452 (100.0)**	
			*P = 0*.*052*				*P<0*.*001*					
3.	**Marital status**	**Unmarried**	83 (62.9)	14 (10.6)	10 (7.6)	**107 (81.1)**	21 (15.9)	4 (3.0)	0 (0.0)	**25 (18.9)**	**132 (100.0)**	**0.148**
		**Ever Married**	106 (21.9)	159 (32.8)	105 (21.6)	**370 (76.3)**	34 (7.0)	55 (11.3)	26 (5.4)	**115 (23.7)**	**485 (100.0)**	
			*P<0*.*001*				*P<0*.*001*					
4.	**Highest qualification**	**MBBS**	73 (60.3)	14 (11.6)	14 (11.6)	**101 (83.5)**	13 (10.7)	4 (3.3)	3 (2.5)	**20 (16.5)**	**121 (100.0)**	**0.293**
		**Specialization (MD/MS/DNB/Diploma)**	103 (24.6)	126 (30.1)	83 (19.8)	**312 (74.5)**	40 (9.5)	45 (10.7)	22 (5.3)	**107 (25.5)**	**419 (100.0)**	
		**Super specialization (DM/MCH/FNB)**	13 (16.9)	33 (42.9)	18 (23.4)	**64 (83.1)**	2 (2.6)	10 (13.0)	1 (1.3)	**13 (16.9)**	**77 (100.0)**	
			*P<0*.*001*				*P = 0*.*071*					
6.	**Years of experience**	**<5 years**	95 (51.9)	30 (16.4)	15 (8.2)	**140 (76.5)**	24 (13.1)	14 (7.7)	5 (2.7)	**43 (23.5)**	**183 (100.0)**	**0.455**
		**5–10 years**	63 (31.0)	52 (25.6)	51 (25.1)	**166 (81.8)**	16 (7.9)	12 (5.9)	9 (4.4)	**37 (18.2)**	**203 (100.0)**	
		**11–15 years**	11 (15.3)	26 (36.1)	19 (26.4)	**56 (77.8)**	7 (9.7)	4 (5.6)	5 (6.9)	**16 (22.2)**	**72 (100.0)**	
		**16–20 years**	12 (18.2)	29 (43.9)	11 (16.7)	**52 (78.8)**	4 (6.1)	7 (10.6)	3 (4.5)	**14 (21.2)**	**66 (100.0)**	
		**>20**	8 (8.6)	36 (38.7)	19 (20.4)	**63 (67.7)**	4 (4.3)	22 (23.7)	4 (4.3)	**30 (32.3)**	**93 (100.0)**	
			*P<0*.*001*				*P = 0*.*003*					
7.	**Area of practice**	**Village**	24 (50.0)	4 (8.3)	8 (16.7)	**36 (75.0)**	6 (12.5)	3 (6.3)	3 (6.3)	**12 (25.0)**	**48 (100.0)**	**0.484**
		**Town/city**	119 (28.3)	124 (29.5)	76 (18.1)	**319 (76.0)**	38 (9.0)	47 (11.2)	16 (3.8)	**101 (24.0)**	**420 (100.0)**	
		**Metropolitan city**	46 (30.9)	45 (30.2)	31 (20.8)	**122 (81.9)**	11 (7.4)	9 (6.0)	7 (4.7)	**27 (18.1)**	**149 (100.0)**	
			*P = 0*.*005*				*P = 0*.*410*					

*p-value for comparison between study “total participants faced workplace violence” and “total participants not faced workplace violence”.

On subgroup analysis, it was seen that the distribution of DWPV according to the place of work (government and/or private) was significantly associated with age, marital status, highest qualification, years of experience, and area of practice. It was also observed that study participants with higher experience, qualification, and those who were ever-married were more likely to work in privately owned health institutions, especially those located in urban areas. The details and comparison between the two groups have been given in **Table 1**.

### Type of violence and its reporting pattern

Overall, verbal abuse (91.2%) was the most common type of violence faced by the DWPV, followed by verbal threat (60.8%). One hundred and seventeen (24.5%) doctors had faced violence in the one week before filling the form, 40.0% DWPV had faced violence between one week to one month and 78.8% of the DWPV faced violence from one month to one year before filling the form. All 477 doctors had experienced some type of WPV at different points of time before one year of filing the Google form. Around half of the DWPV reported the incidents to their senior authorities. Sexual violence was the least reported type of WPV—with 91.3% of cases never reported. Only the incidents of major physical injury were reported officially at all times. The distribution of DWPV according to the type of WPV faced and its’ reporting, is depicted in **Table 2**.

**Table 2:** Distribution of study participants as per the type of WPV faced and it’s reporting, in different periods.

Sl. no.		Last one week before filling the form n (%)		One week to One month before filling the form n (%)		One month to One year before filling the form n (%)		More than one year before filling the form n (%)	
**Workplace violence faced (N = 477)**									
**1.**	**Did not face any type of workplace violence**	360 (75.5)		286 (60.0)		101 (21.2)		0 (0.0)	
**2.**	**Faced any type of workplace Violence**	117 (24.5)		191 (40.0)		376 (78.8)		477 (100.0)	
**Workplace violence reported**									
		**Did not report**	**Report**	**Did not report**	**Report**	**Did not report**	**Report**	**Did not report**	**Report**
**1.**	**Verbal abuse (VA)**	20 (35.1)	30 (50.0)	38 (44.2)	44 (41.9)	65 (42.2)	87 (39.2)	72 (32.6)	79 (30.9)
**2.**	**Verbal threat (VT)**	10 (17.5)	10 (16.7)	19 (22.1)	31 (29.5)	42 (27.3)	66 (29.7)	59 (26.7)	53 (20.7)
**3.**	**Intimidation (IN)**	13 (22.8)	08 (13.3)	17 (19.8)	13 (12.4)	26 (16.9)	25 (11.3)	34 (15.4)	29 (11.3)
**4.**	**Minor physical injury (PI)**	05 (8.8)	01 (1.7)	01 (1.2)	01 (1.0)	11 (7.1)	18 (8.1)	24 (10.9)	35 (13.7)
**5.**	**Major physical injury (PI)**	0 (0.0)	05 (8.3)	0 (0.0)	06 (5.7)	0 (0.0)	09 (4.1)	0 (0.0)	23 (9.0)
**6.**	**Sexual violence (SV)**	06 (10.5)	0 (0.0)	04 (4.7)	0 (0.0)	02 (1.9)	0 (0.0)	14 (6.3)	02 (0.8)
**7.**	**Damage to physical property (PD)**	03 (5.2)	06 (10.0)	07 (8.1)	10 (9.5)	08 (5.2)	17 (7.7)	18 (8.1)	35 (13.7)
	**Total**	**57 (100.0)**	**60 (100.0)**	**86 (100.0)**	**105 (100.0)**	**154 (100.0)**	**222 (100.0)**	**221 (100.0)**	**256 (100.0)**

### Reporting authority and satisfaction with redressal

Approximately half (52.1%) of the incidents of WPV were reported to seniors and/or head of the departments—that too mostly verbally, 32.8% incidents were reported to the administrators, and in only 24.5% of the incidents the complaint was lodged with the police. A small number (9.9%) of the study participants, who officially reported the incident of violence, were redressed satisfactorily, 39.2% got redressed to some extent and in 26.8% of cases, no amends were done. The incident of violence was shared on electronic media and social platforms by 17.2% of the DWPV out of which 11.2% were shared on WhatsApp, 8.9% on Facebook, and only 0.5% incidents were shared in mass media.

### Why did DWPV not report the incident to the competent authority?

Out of 477 DWPV, 177 (37.1%) cited reasons for not reporting the incidents of WPV. The perception of “no action will be taken” against the perpetrators (21.5%) was the commonest reason found in our study. Approximately one-fifth of the incidents (20.9%) were resolved at the local level. Another one-fifth of the respondents (20.3%) believed that reporting the incidents of violence was more stressful and time-consuming, and also causes negative publicity. Another one-fifth DWPV (19.8%) ignored the incident considering WPV as normal and part of the job, and; 11.3% overlooked the incidents because those were not of significant severity for them [VA and (VT + IN]. Some of the incidents were not reported because of the indifferent and/or hostile attitude of the administrators and the police. A minor number of DWPV did not report due to political influence/interference (5.1%), and; 4.0% of the DWPV were not aware of the procedure of reporting.

### Factors associated with severity of violence

In the present study, WPV was found to be significantly associated with the gender of DWPV (p = 0.015), with male doctors seen to be more prone to different types of WPV. No significant association was observed between other socio-demographic and professional variables with the type and severity of WPV faced (**Table 3**).

**Table 3:** Distribution of Workplace violence as per socio-demography and professional details of study participants.

S. no.	Variables	Groups	VA only	(VT + IN) ± VA	(PI + SV + PD) ± VA ± (VT + IN)	p-value
			N = 148 n (%)	N = 210 n (%)	N = 119 n (%)	
1.	**Age (years)**	≤30	41 (27.7)	47 (22.4)	33 (27.7)	0.848
		31–40	58 (39.2)	96 (45.7)	45 (37.8)	
		41–50	36 (24.3)	45 (21.4)	27 (22.7)	
		51–60	8 (5.4)	14 (6.7)	10 (8.4)	
		>60	5 (3.4)	8 (3.8)	4 (3.4)	
2.	**Gender**	Female	51 (34.5)	48 (22.9)	24 (20.2)	0.015
		Male	97 (65.5)	162 (77.1)	95 (79.8)	
3.	**Marital status**	Unmarried	31 (21.0)	45 (21.4)	31 (26.0)	0.555
		Ever married	117 (79.0)	165 (78.6)	88 (74.0)	
4.	**Highest qualification**	MBBS	29 (19.6)	38 (18.1)	34 (28.6)	0.247
		Specialization (MD/MS/DNB/Diploma)	100 (67.6)	143 (68.1)	69 (58.0)	
		Super specialization (DM/MCH/FNB)	19 (12.8)	29 (13.8)	16 (13.4)	
6.	**Years of experience**	<5 years	51 (34.5)	55 (26.2)	34 (28.6)	0.505
		5–10 years	46 (31.1)	82 (39.0)	38 (31.9)	
		11–15 years	16 (10.8)	26 (12.4)	14 (11.8)	
		16–20 years	18 (12.2)	19 (9.1)	15 (12.6)	
		>20	17 (11.5)	28 (13.3)	18 (15.1)	
7.	**Area of practice**	Village	6 (4.1)	18 (8.6)	12 (10.1)	0.184
		Town/city	101 (68.2)	135 (64.3)	83 (69.8)	
		Metropolitan city	41 (27.7)	57 (27.1)	24 (20.1)	
8.	**Type of health facility working**	Government Health Facility	52 (35.1)	86 (41.0)	51 (42.9)	0.886
		Own private clinic	8 (5.4)	13 (6.2)	7 (5.9)	
		Non-government / private hospitals	49 (33.1)	61 (29.0)	35 (29.4)	
		Multiple	39 (26.4)	50 (23.8)	26 (21.9)	

VA: Verbal Abuse, VT: Verbal Threat, IN: Intimidation, PI: Physical Injury, SV: Sexual Violence, PD: Property Damage.

### The perceived causes of violence

The most common reported cause of the violence was “actual or perceived non-improvement or deterioration of the patient’s condition” (40.0%). This was followed by “perception of wrong treatment given” (37.3%), “death of the patient” (34.4%), and “actual or perceived delay in treatment” (28.5%). Other causes of violence cited were- “unrealistic demands from patient and patient party” such as issuing a false certificate, early discharge, special preferences, etc. (9.2%); cost and fee-related issues (5.0%); and reasons associated with administrative failure and poor infrastructure, like, long waiting time, unavailability of bed, drugs, investigations, etc. (3.4%).

### The perpetrators of violence

In the majority of the incidents of violence, family members and/or relatives of the patient were the main perpetrators (82.2%). Friends of the family were involved in 44.4% of the incidents. In 14.9% of cases, the patient himself/herself caused violence. Onlookers were responsible for 12.4% of cases and in 4% of cases political leaders were reported to be the perpetrators. Sixteen percent of the study participants reported that the perpetrators were under the influence of alcohol at the time of the incident and 21.1% of the cases the DPWV suspected that the perpetrators were under influence of alcohol.

### The psycho-social impact of the severity of violence on the DWPV

More than half of the DWPV reported “loss of self-esteem & feeling of shame” (52.2%), and “stress/depression/anxiety/ideas of persecution” (51.2%). Around half of the DWPV felt a sense of defeat (41.7%) while giving their best in the profession. Fifty-nine (12.4%) study participants said that they had to change their place of work and shift to another place/town after the incident (**Table 4**).

**Table 4:** Distribution of Psycho-social impact of workplace violence by the type of violence faced by the study participants.

S. no.	Type of Violence Psycho-social Impact*	VA only N = 148 n (%)	(VT + IN) ± VA N = 210 n (%)	(PI + SV + PD) ± VA ± (VT + IN) N = 119 n (%)	TOTAL N = 477 n (%)
1.	Loss of self-esteem and shame	61 (41.2)	108 (51.4)	80 (67.2)	249 (52.2)
2.	Stress/depression/anxiety/idea of persecution	45 (30.4)	117 (55.7)	82 (68.9)	244 (51.2)
3.	Sense of defeat	42 (28.4)	92 (43.8)	65 (54.6)	199 (41.7)
4.	Increased aggressiveness towards patients	46 (31.1)	75 (35.7)	45 (37.8)	166 (34.8)
5.	Avoidance/missing work & Loss of productivity and income	20 (13.5)	66 (31.4)	47 (39.5)	133 (27.9)
6.	Avoiding social gatherings/Social disruption	6 (4.1)	29 (13.8)	24 (20.2)	59 (12.4)
7.	Had to change place of work/shift to another place	8 (5.4)	28 (13.3)	23 (19.3)	59 (12.4)
8.	Engaging in risky behaviors & substance use	0 (0.0)	13 (6.2)	9 (7.6)	22 (4.6)
9.	No impact	8 (5.4)	3 (1.4)	1 (0.01)	12 (2.5)
**Trauma Screening Questionnaire**					
1.	Score <6	127 (85.8)	147 (70.0)	70 (58.8)	344 (72.1)
2.	Score ≥6	21 (14.2)	63 (30.0)	49 (41.2)	133 (27.9)

*Multiple response.

As per Trauma Screening Questionnaire (TSQ) [15], presence of impact of violence on DWPV was found to be 2.6 (1.5–4.7) times higher in cases of (VT + IN) ± VA and 4.2 (2.3–8.0) times higher in cases of (PI+SV+PD) ± VA ± (VT + IN) than DWPV who faced verbal abuse only.

### Impact of severity of violence on patient management

The incidents of WPV had a significant impact on patient management and decision making by the treating doctor. Management by surgical and medical interventions and handling of emergency/critical/complicated cases decreased with an increase in the severity of violence against doctors. On the contrary, suggesting investigations and referrals along with consultation with other specialists were reported to be increased. However, there was no change in the handling of non-complicated/non-emergency cases (**Table 5**).

**Table 5:** Impact of severity of violence on patient management and decision making by the doctors who faced workplace violence.

S. no.	Impact on patient management		Severity of Violence			p-value
			VA only N = 148 n (%)	(VT + IN) ± VA N = 210 n (%)	(PI + SV + PD) ± VA ± (VT + IN) N = 119 n (%)	
1.	**Prescribing Drugs**	Same as before	109 (73.6)	140 (66.7)	75 (63.0)	0.069
		Decreased	20 (13.5)	26 (12.4)	25 (21.0)	
		Increased	19 (12.8)	44 (21.0)	19 (16.0)	
2.	**Surgical or Medical interventions**	Same as before	106 (71.6)	111 (52.9)	46 (38.7)	<0.001
		Decreased	26 (17.6)	72 (34.3)	62 (52.1)	
		Increased	16 (10.8)	27 (12.9)	11 (9.2)	
3.	**Suggesting investigations**	Same as before	90 (60.8)	90 (42.9)	40 (33.6)	<0.001
		Decreased	19 (12.8)	25 (11.9)	22 (18.5)	
		Increased	39 (26.4)	95 (45.2)	57 (47.9)	
4.	**Handling emergency/critical cases**	Same as before	63 (42.6)	47 (22.4)	30 (25.2)	0.001
		Decreased	76 (51.4)	145 (69.0)	79 (66.4)	
		Increased	9 (6.1)	18 (8.6)	10 (8.4)	
5.	**Handling non-complicated cases**	Same as before	99 (66.9)	133 (63.3)	70 (58.8)	0.424
		Decreased	22 (14.9)	41 (19.5)	29 (24.4)	
		Increased	27 (18.2)	36 (17.1)	20 (16.8)	
6.	**Referral/Consultation liaison**	Same as before	74 (50.0)	55 (26.2)	22 (18.5)	<0.001
		Decreased	15 (10.1)	10 (4.8)	15 (12.6)	
		Increased	59 (39.9)	145 (69.0)	82 (68.9)	

## Discussion

It is a great irony that medicine, the epitome of the healing profession, has been strained with violence. Healthcare WPV has emerged as a serious threat to the doctor-patient relationship all over the world. The actual extent of the problem is estimated to be substantially bigger, as WPV remains grossly neglected and under-reported [18]. Much remains to be done for proper reporting, documentation, and prevention/control of WPV against doctors. Identification of the risk factors is very important to prevent and predict such incidents and restore the faith and trust of society towards medical fraternity. In this study, we have tried to identify the risk factors related to WPV against doctors and their complex interaction with patient management in the Indian scenario.

### Potential risk-factors of WPV related to the doctor

In the present nationwide cross-sectional study, we found the frequency of WPV is high both in government and private healthcare settings and almost equal in frequencies across all states. Male unmarried physician below the age of 30 years was found to be at higher risk. This may be due to more exposure and less experience of young doctors, as was also reported in other studies [19–21]. On the other hand, it may be argued that female, and an aged male doctor may be better in managing potentially problematic situations. Similar to other studies, doctors with higher educational degrees were found to be more prone to violence, in this study [22]. This may be the result of higher expectations and handling of more complicated cases by the specialists. Also, patients typically assume that doctors with a higher educational degree have better medical capabilities and skills; and if the outcomes do not meet patient’s expectations, chances of disputes increase.

Interestingly, we found that the duration of experience did not play a significant role in the prevention of WPV in India. This is not consistent with other studies [23], but various incidents of WPV reported in Indian media involving the death of a senior health official and grievous injury of junior doctors provide evidence for the same. Though no significant difference was found, participants from urban areas reported more occurrence of WPV than rural areas. This is in line with other studies that have reported that doctors from urban settings are more vulnerable to WPV [24]. This is probable, as the concentration of doctors and thereby patients are much higher in urban areas in absolute numbers; also, the secondary- and tertiary- level health facilities are usually located in urban areas, which get to handle a greater number of complicated cases with higher morbidity and mortality.

### Characteristics of perpetrators of WPV

Family members/relatives (82.2%), family friends (4.4%), and the patient himself/herself (14.9%) were the main perpetrators of WPV, in our study. The same finding was also reported by other studies [25, 26]. The probable reason suggested is that the patients’ relatives and the patient himself/herself are in a lot of physical, psycho-social, and economic stress during the disease, which exacerbated by the lack of infrastructure gives rise to dissatisfaction, thereby leading them to commit violence against the medical personnel. We found that 16% of the perpetrators were under the influence of alcohol. The primary purpose of profiling of offenders is to identify persons with a higher tendency to cause workplace violence so that safeguards can be implemented to prevent violence before it occurs. At the policy level, measures must be incorporated into existing occupational health and safety legislation to formulate and implement strategies to manage people accompanying the patient, e.g., strategy for stricter security, controlled access, registration, and identification at the front desk, for better prevention and control of WPV [11].

### Types of violence

We found verbal abuse (91.2%) and verbal threats (60.8%) are common and consistent forms of violence faced by physicians. Studies across the globe have also reported non-physical violence as the commonest type of violence face by doctors [21, 27]. Arguably, in the present study, a greater number of study participants reported facing verbal threats and intimidation rather than verbal abuse. This is a very disturbing trend of WPV in India and needs urgent action; as it has been known that verbal threat and intimidation may lead to a criminal act in the future. One such documented form of organized WPV is “Yi Nao” in China [28], and; such cases have been hypothesized to be on an increase in India [29].

### Perceived causes of violence

Actual or perceived—non-improvement or deterioration of patient’s condition (40.0%),—wrong treatment (37.3%), and death of the patient (34.4%) were some of the main causes of WPV stated in our study. Unrealistic demands (9.2%), cost, and fee-related issues (5.0%) were other minor reasons for violence, in our study. A study conducted among second- and third-year resident doctors working in three teaching hospitals in Northern India reported that non-availability of medicine, dissatisfaction with service; death, and referral of the patients were the main reasons for WPV [25]. Another study conducted among primary health care physicians working in Karachi, Pakistan reported that long waiting time and dissatisfaction with treatment were common causes for violence against doctors [2]. The general population has developed a perception that doctors are corrupt and indulging in unethical practices; this distrust for doctors in the community has been fuelled by the flagrant media reporting presenting whole medical fraternity on the wrong foot. As a result, the community has lost faith in doctors and all negative outcomes of treatment are being portrayed as negligence and/or fault of doctors. Also, ‘googling’ medical symptoms and its management, from non-authentic sites, providing incomplete understanding, has increased the perception of wrong treatment and, thereby, increased dissatisfaction among patients/relatives.

Globally, it has been reported that in recent years the society has become more intolerant and more inclined towards violent activities [30]. Our study findings provide a snapshot of this deteriorating social fabric. These facts instigate some crucial steps for prevention of WPV, e.g., healthcare providers taking informed and valid consent from the patients and/or their family members after a detailed explanation about the prognosis, treatment outcome, the need for surgery, and cost of the treatment in the language and dialect of the patients. For these, the communication skills of doctors have to be improved. Even though medical practitioners in their forties and fifties may have formulated their methods to communicate with patients, they keep up with the times and train in communication skills from experts as part of their continuing medical education. The Medical Council of India (MCI) has recently emphasized incorporating communication skills in the MBBS curriculum; however, the medical institution needs to train medical undergraduates on this skill more religiously so that young doctors develop this essential and important skill [31].

### Risk-factors related to the workplace

According to the National Health Profile 2019, the government expenditure on health is around 1.3% of the country’s total GDP [32]. The National Sample Survey (NSS) 2014 estimated that more than 70% of spells of ailment were treated in the private sector with a preference towards allopathic treatment [14]. The private sector consists of private practitioners, for-profit hospitals and nursing homes, and charitable hospitals—they are diverse and fragmented. In India, 33% of healthcare expenditure is from government sources, and the remaining, majority is out-of-pocket expenditure [33]. The cost of medical treatment has skyrocketed over the yearsand this out-of-pocket expense for healthcare pushes many households into poverty. The meager government expenditure in public healthcare is, consequently, leading to a wide gap in supply and demand in terms of infrastructure, manpower and facilities in government healthcare settings which in turn makes doctors exposed to an unmanageable number of patientsresulting in a delay in treatment, unexpected cost, long waiting time, unavailability of bed, drugs, investigations, etc. [14]. The frustration and anxiety among family members, when the treatment outcome becomes unfavourable, gets intensified with the above-mentioned consequences of low government expenditure and low health insurance coverage in India [34], which in turn escalates the possibility of violence against doctors.

### Psychosocial impact

WPV against doctors has an immense psycho-social impact. More than half of the victims reported “stress/depression/anxiety/ideas of persecution” and “loss of self-esteem and shame” after the incidents. The psychological impact of these traumatic events of violence increased significantly with the severity of trauma faced by the study participants; the findings of TSQ suggest that a large number of doctors who faced workplace violence need specialist psychological help and counselling [15]. In India, the doctor-patient ratio is already very low which results in heavy workload among medical professionals [35]. This when combined with a depressed and anxious workforce of doctors, further worsens the health-care service provision in the country. Some studies in India have reported that doctors are often stressed and suffer from mental disorders which many times lead to suicide [36, 37]. If violence against doctors becomes rampant, it would affect the already stressed community tremendously and would ultimately have a ripple effect.

### Effect of WPV on patient management

WPV does not only have a psycho-social impact on doctors, but it also has a repercussion effect on decision making by doctors. Our study reported that “surgical and medical interventions” and handling of emergency cases were inversely related to the severity of violence experienced by the treating doctor. On the other hand, “prescribing investigations” and “referrals” along with consultation from other doctors increased. In both cases, it is evident that WPV against doctors would increase unnecessary referrals. This would not only add to the already existing high burden of patients in higher centers but also create inconvenience to the patients. An increase in prescribing investigations, while not utilizing clinical acumen, by doctors, would increase the cost of treatment, and consequently have a heavy impact on already resource constraint health care setting.

**Fig 2:**
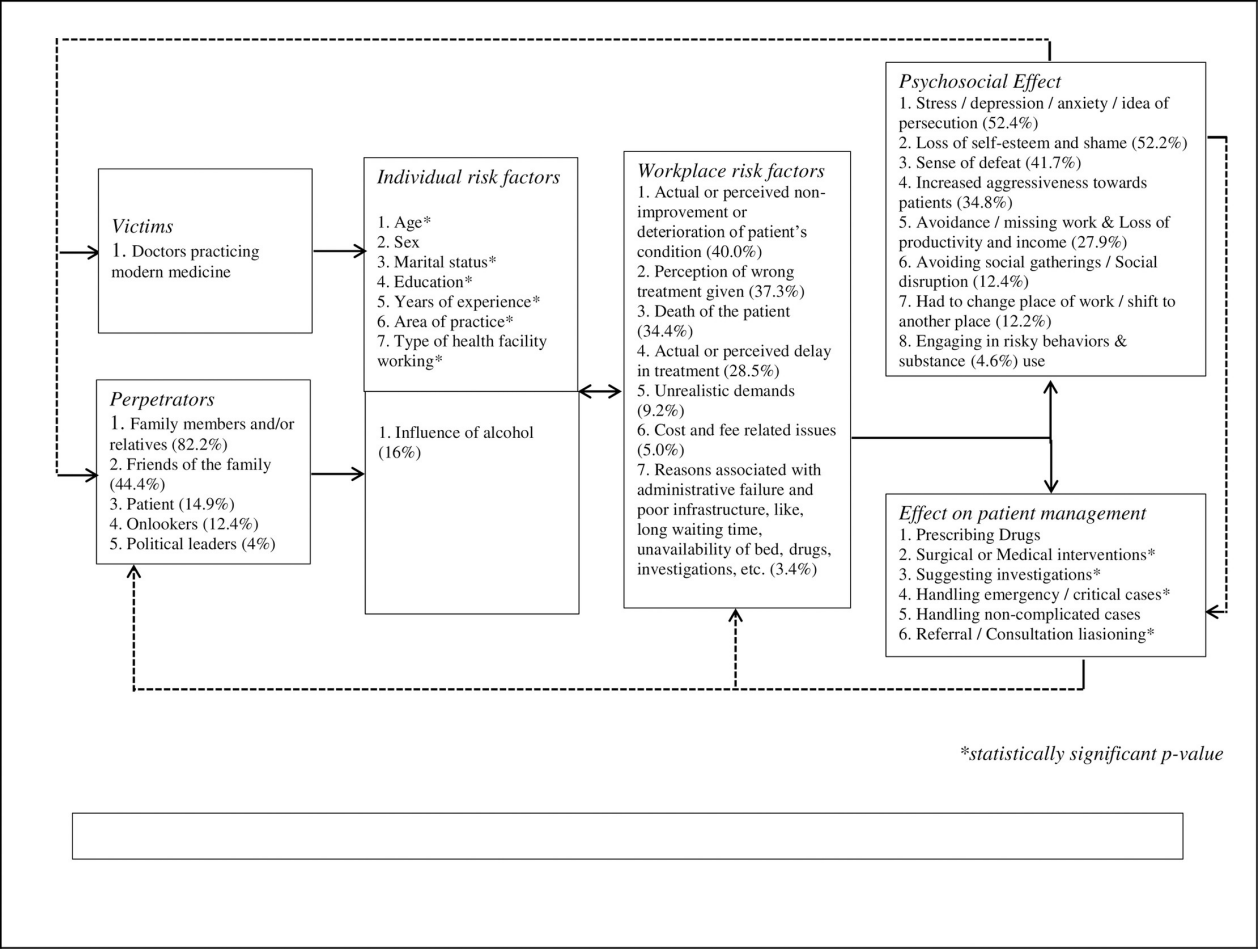
Model framework on workplace violence against doctors; based on Chappell and Di Martino’s interactive model (2006).

From the above discussion, it is evident that multiple risk factors are contributing to violence at the workplace against doctors. These risk factors have an individual effect on violence, as well as contribute in an interactive and dependent manner. Understanding the impact of all the relevant risk factors in generating violence at the workplace is important in the perspective of doctors as this will help in planning appropriate strategies for the prevention of the problem. In Fig 2, we have explained the interaction of multiple risk factors and their contribution to the incidence of workplace violence with Chappell di Martino’s model [38].

### Synthesis of the model framework on workplace violence against doctors and recommendations based on Chappell and Di Martino model

For identification of important risk factors and to understand the interaction of multiple factors related to WPV against doctors we have created a model based on Chappell and Di Martino model (Fig 2). Chappell and Di Martino model was used and has proved useful in the prevention of healthcare-related WPV by Cristina Vidal-Martía et al. [38].

From this model (Fig 2), it is evident that more than 50% of the physician suffers from stress/depression/anxiety/idea of persecution, loss of self-esteem, and shame as a result of WPV. Subsequently, patient management is hampered, especially surgical and complicated cases, which may face more referral and costly investigation. This, in turn, may escalate discontent among people accompanying the patient and thereby, increase in the workplace violence risk factors like delay in treatment and deterioration in a patient’s condition. The whole situation becomes a never-ending, self-propagating vicious cycle as depicted in the model. To break this cycle, attention should be given to develop and implement measures for addressing the stress among doctors due to WPV. Appropriate strategies of documentation and stringent legal action are needed to predict and control such incidents. The problem with the present law is that after violence has occurred, the doctor files a police complaint and simultaneously the patient and relatives file a complaint about criminal negligence. This results in a compromise between two parties and no punishment to the perpetrators; hence, no deterrence for the next incidence of violence.

### Limitations and strengths

To achieve the target sample-size we adopted a non-probability sampling technique, which may compromise the representativeness of the sample. However, there was no statistically significant difference between respondent doctors who did and did not face WPV in terms of socio-demographic and professional characteristics. This observation was important as this showed that though we adopted convenient sampling, the two groups were not statistically different. This has reduced the chance of selection bias and ensured the internal validity of our study.

As the present study involved data collection through Google forms, so medical fraternity who were not accustomed to using new technology, especially the senior doctors, might not have been able to give their responses. There might have been some recall bias as some of the questions required information from more than a year duration. Also, in the present study, we were not able to estimate the number of incidents of WPV faced by each doctor.

This is the first study conducted in India which represents medical fraternity from almost all states and Union Territories. It is also one of its kind studies to have a good representation of doctors working in both the private sector and government health facilities. In the present study, we have included the impact of workplace violence on patient management, which has not been documented yet in India.

## Conclusion

WPV is a significant problem for doctors in India. In our study, a large number of members of medical fraternity reported facing WPV, irrespective of age, gender, place of work, experience, and professional expertise. At most times, these incidents of violence are in the form of verbal abuse, verbal threat, and intimidation; with meager reporting of incidents and unsatisfactory redressal. Actual or perceived non-improvement or deterioration of a patient’s condition with delay in treatment, perception of wrong treatment given, and death of the patient are important factors contributing to violence. Lack of trust fuelled by concocted news in media against the doctor community reflects a picture of social degradation, intolerance, and crisis. The unsafe workplace is affecting the system in two folds–it is hampering the psycho-social health of doctors and also, altering their patient management practices–with more doctors adopting a safer approach to avoid WPV. The ultimate brunt is not only borne by the medical fraternity who are targeted, but also by the general public who don’t get expected healthcare.

For mutual benefit and betterment of society at large, stringent workplace policies and procedures are needed especially on reporting of violence and subsequent surveillance. Regular and continuous medical education program on policies regarding prevention and management of violence should be done from a common platform, like, Indian Medical Association, for all practising doctors. This common platform may be used to offer debriefing and counselling when violence occurs. Medical education can further incorporation of necessary communication skills as an integral part of the medical curriculum. Media has to be sensitised for developing mutual understanding between the medical fraternity and the general public. Lastly, the implementation of laws has to be ensured to rightly punish any culprits and thereby, strengthen trust in the judicial system. The doctor-patient relationship and trust is the major backbone for the public health of India. All efforts should be taken to restore trust, and future research should be conducted to identify best practices for preventing violence.

## Supporting information

S1 Questionnaire (DOCX)
