# Supplementary material for: Pattern of workplace violence against doctors practising modern medicine and the subsequent impact on patient care, in India
Source: PLoS One. 2020 Sep 18;15(9):e0239193. doi: 10.1371/journal.pone.0239193 (PMC7500628; doi:10.1371/journal.pone.0239193)
Supplement: S1 Questionnaire — (DOCX) [file pone.0239193.s001.docx]

**Violence against doctors and its impact on patient management: An internet based survey.**

**Section 1**

1. AGE (in completed years)………………….
2. SEX –
   1. Male
   2. Female
3. MARITAL STATUS –
   1. Married
   2. Unmarried
   3. Separated or divorced
   4. Widowed
4. QUALIFICATION (MARK THE HIGHEST QUALIFICATION) –
   1. MBBS
   2. MD/MS
   3. Diploma
   4. DM/MCh
   5. DNB
5. DEPARTMENT / SPECIALITY / SUPER-SPECIALITY PRACTICING IN…………………
6. YEARS OF EXPERIENCE / PRACTICE (in completed years)…………………..
7. STATE IN WHICH WORKING / PRACTICING………………….
8. MAJOR AREA IN WHICH WORKING / PRACTICING
   1. Village
   2. Town
   3. City
   4. Metropolitan city
9. PLACE OF WORK (multiple answers possible)
   1. Corporate Hospital
   2. Private Hospital / Nursing home
   3. Own Private Clinic
   4. Chamber with chemist and / or polyclinic
   5. Government Medical College
   6. District / Sub-District Hospital
   7. CHC / PHC
10. Have you ever experienced any type of Violence during practice (Verbal or Physical or damage to property - minor or major)
    1. Yes – (Go to Section 2)
    2. No – (Go to Section 3)

**IF EXPERIENCED VIOLENCE - SECTION 2**

1. Type of Violence experienced (multiple answers possible)
   1. Verbal Abuse
   2. Verbal Threat - to self-and/or family
   3. Minor Physical Injury - not requiring specialist medical help
   4. Major Physical Injury / Assault
   5. Sexual violence
   6. Intimidation - forced to do something against your wish
   7. Damage to physical property
2. When did you last face violence? (Answer according to the type of violence experienced; mention "Not Applicable" in other cases)

|  | Not Applicable | Within Last week | In last month | Within last one year | Any time before one year |
| --- | --- | --- | --- | --- | --- |
| Verbal Abuse  (personally attacked, devalued or humiliated via the spoken word) |  |  |  |  |  |
| Verbal Threat  (warnings with the intent to injure) |  |  |  |  |  |
| Minor Physical Injury – required no or minimal medical attention |  |  |  |  |  |
| Major Physical Injury / Assault |  |  |  |  |  |
| Sexual violence |  |  |  |  |  |
| Intimidation - forced to do something against your wish |  |  |  |  |  |
| Damage to physical property |  |  |  |  |  |

1. What was the reason of violence? (multiple answers possible)
   1. Death of patient
   2. Delay in treatment
   3. Patient condition did not improve / worsen
   4. Perception of wrong treatment given
   5. Other……………….
2. Who were the Perpetrators (people committing the act) of violence? (multiple answers possible)
   1. Patient himself / herself
   2. Family members / Relatives
   3. Friends of the family
   4. On lookers
   5. Others………………..
3. Was the person inciting violence under influence of alcohol and / or drugs?
   1. Yes
   2. No
   3. Maybe
4. Did you tell about / report the incident? (Answer according to the type of violence experienced; mention "Not Applicable" if not experienced that type of violence and "Every time" even if in single incident)

|  | Not Applicable | Never | Few times | Every time |
| --- | --- | --- | --- | --- |
| Verbal Abuse |  |  |  |  |
| Verbal Threat |  |  |  |  |
| Minor Physical Injury - not requiring specialist medical help |  |  |  |  |
| Major Physical Injury / Assault |  |  |  |  |
| Sexual violence |  |  |  |  |
| Intimidation - forced to do something against your wish |  |  |  |  |
| Damage to physical property |  |  |  |  |

1. Did you report the incident to competent authority / senior? (multiple answers possible)
   1. No
   2. To my seniors / Head
   3. To the administrators
   4. To the police
   5. Other……………
2. Did you get any help or redressal after reporting the incident to competent authority / senior?
   1. Not Applicable
   2. No
   3. To some extent
   4. Satisfactorily
3. If the incident was NOT OFFICIALLY REPORTED to competent authority / senior - PLEASE SPECIFY THE REASON of not reporting the incident of violence against you ...................
4. Was the incident shared in social media? (multiple answers possible)
   1. No
   2. Facebook
   3. WhatsApp
   4. Other…………………..
5. How did the incident of violence affect any of the following - Your..........

|  | Increased | Decreased | Same as before |
| --- | --- | --- | --- |
| Prescribing drugs |  |  |  |
| Surgical or medical interventions |  |  |  |
| Suggesting investigations |  |  |  |
| Handling emergency / critical / complicated cases |  |  |  |
| Handling non-complicated cases |  |  |  |
| Referral / Consultation liasioning with other specialists |  |  |  |

1. What psycho-social impact did the violence have on you? (multiple answers possible)
   1. Sense of defeat
   2. Loss of self-esteem & shame
   3. Avoidance/Missing work & Loss of productivity & income
   4. Engaging in risky behaviours & substance use
   5. Stress / depression / anxiety / persecutional ideas
   6. Avoiding social gatherings / Social disruption
   7. Increased aggressiveness towards patients
   8. Had to change place of work/shift to other place
   9. Other………………
2. Trauma Screening Questionnaire

|  | YES (at least twice in the past week) | No |
| --- | --- | --- |
| Upsetting thoughts or memories about the event that have come into your mind against your will |  |  |
| Upsetting dreams about the event |  |  |
| Acting or feeling as though the event were happening again |  |  |
| Feeling upset by reminders of the event |  |  |
| Bodily reactions (such as fast heartbeat, stomach churning) |  |  |
| Difficulty falling or staying asleep |  |  |
| Irritability or outbursts of anger |  |  |
| Difficulty concentrating |  |  |
| Heightened awareness of potential dangers to yourself and others |  |  |
| Feeling jumpy or being startled by something unexpected |  |  |
